# Neuropeptide FF receptor 2 overexpression aggravates lipid accumulation and metabolic dysfunction in mice with diet-induced metabolic stress

**DOI:** 10.1016/j.bj.2025.100913

**Published:** 2025-09-12

**Authors:** Hsiang-Ting Hsu, Chun-Chun Hsu, Yun-Jou Liao, Hui-Yun Li, Yao-Chang Chiang, Ya-Tin Lin

**Affiliations:** a Department of Medicine, Chang Gung University, Taoyuan, Taiwan; b School of Respiratory Therapy & Graduate Institute of Medical Sciences, College of Medicine, Taipei Medical University, Taipei, Taiwan; c Division of Pulmonary Medicine, Department of Internal Medicine, Taipei Medical University Hospital, Taipei, Taiwan; d Graduate Institute of Metabolism and Obesity Sciences, College of Nutrition & TMU Research Center for Digestive Medicine, Taipei Medical University, Taipei, Taiwan; e Department of Natural Sciences, Oregon Institute of Technology, Klamath Falls, OR, USA; f Chronic Diseases and Health Promotion Research Center & Department of Nursing, Division of Basic Medical Sciences, Chang Gung University of Science and Technology, Chiayi County, Puzi City, Taiwan; g Nutrition Research Center, Taipei Medical University Hospital, Taipei, Taiwan

**Keywords:** NPFF, NPFFR2, Obesity, Adipocyte hypertrophy, Glucose homeostasis

## Abstract

**Background:**

Obesity is a major contributor to metabolic dysfunction and is driven by complex genetic, behavioral, and physiological factors. Neuropeptide FF receptor 2 (NPFFR2) has been implicated in regulating feeding behavior, as well as energy and glucose homeostasis. However, its precise role in obesity and metabolic disorders remains unclear. This study aimed to investigate the systemic role of NPFFR2 in obesity-induced metabolic dysfunction.

**Material and methods:**

The role of NPFFR2 was examined using wild-type and *Npffr2*-overexpressing transgenic mice subjected to 15 weeks of high-fat high-sucrose diet to induce obesity. Systemic, tissue-specific, and serum metabolic profiles were analyzed, with a particular focus on lipid abnormalities in the liver and adipose tissues.

**Results:**

*Npffr2* overexpression exacerbated obesity-induced metabolic dysfunction, including accelerated body weight gain, impaired glucose homeostasis, altered fat composition, adipose tissue inflammation, and dysregulated lipid metabolism. In addition, hypertrophy of both hepatocytes and adipocytes was aggravated in *Npffr2*-overexpressing mice, collectively contributing to excessive energy storage and reduced metabolic efficiency.

**Conclusions:**

These findings suggest that NPFFR2 may contribute to the regulation of energy balance and lipid metabolism, potentially via central regulatory pathways. These findings highlight the need for mechanistic studies to clarify its region-specific roles and therapeutic potential in metabolic disorders.

## Highlights

• Hypothalamic NPFFR2 overexpression increases fat inflammation and hypertrophy.
• NPFFR2 overexpression promotes energy storage and reduces metabolic efficiency.
• NPFFR2 functions in a context-dependent manner via central and peripheral mechanisms.

## 1 Introduction

Obesity has emerged as a global health crisis, posing significant threats to both human health and economic stability. While prior studies have extensively examined obesity-related risk factors, ranging from individual behaviors and socioeconomic status to environmental influences [1], the precise biological mechanisms driving obesity and the regulatory factors that either promote or mitigate its progression are still under investigation [2]. Multiple genes have been found to play significant roles in obesity development, highlighting the complexity of this physiological condition [3]. Additionally, various physiological mechanisms, such as inflammation, insulin resistance, and central regulation, have been associated with fat accumulation and the resulting metabolic changes [[4], [5], [6], [7]].

Neuropeptide FF receptor 2 (NPFFR2) is activated by a class of RF-amide peptides, including neuropeptide FF (NPFF) and neuropeptide AF (NPAF) [8], which are involved in diverse physiological processes such as nociception, appetite regulation, neuroendocrine function, and adipogenesis [9,10]. NPFFR2 is highly expressed in the hypothalamus, where it is believed to play a key role in regulating energy homeostasis [8,11,12]. It has been reported to influence feeding behavior, diet-induced thermogenesis, obesity development, and glucose homeostasis in rodent models of obesity and diabetes [[13], [14], [15], [16]].

NPFF has been found to co-express with insulin and leptin receptors involved in glucose homeostasis, suggesting that central NPFF may regulate systemic metabolism via a vagus nerve-mediated pathway [13]. Similarly, NPFFR2 is co-expressed with the insulin receptor in the hypothalamic arcuate nucleus (ARC) and has been reported to modulate downstream insulin and leptin signaling within this region under diabetic or obese conditions [14,16]. Furthermore, NPFFR2 is not only co-expressed with the orexigenic peptides neuropeptide Y (NPY) and agouti-related peptide (AgRP) in the ARC, but has also been shown to modulate NPY neuronal activity by reducing intracellular cAMP levels [17]. Our previous findings further revealed that hypothalamic NPFFR2 functions as a negative regulator of insulin signaling, contributing to the disruption of systemic metabolic homeostasis [14].

Beyond its central role, NPFFR2 expression has also been detected in peripheral tissues. Studies in both humans and rodents have reported NPFFR2 expression in several organs, including the heart, kidney, and placenta [9]. However, its expression appears to be limited in the liver, skeletal muscle, and adipose tissues [9,16]. Recent findings indicate that adipose tissue macrophages (ATMs) express both NPFF and NPFFR2, and that circulating NPFF levels are reduced in humans and mice with obesity [18]. The findings suggest that NPFF promotes ATM proliferation, which may in turn influence metabolic pathways linked to obesity [18].

Despite these findings, the role of NPFFR2 in coordinating central and peripheral metabolic regulation remains unclear. Therefore, in this study, we aimed to investigate the effects of *Npffr2* overexpression on metabolic parameters and glucose homeostasis in a diet-induced obesity model, with the goal of evaluating its potential as a therapeutic target for obesity management.

## 2 Material and methods

### 2.1 Experimental animals

In the current study, male C57BL/6JNarl littermates wild-type (WT) and *Npffr2* overexpression transgenic mice (*Npffr2* Tg, +/−) were previously confirmed [19] and utilized in the current study. The mice were bred in an SPF environment at Chang Gung University (AAALAC accreditation, November 2018), maintained at 22 ± 1 °C, 45 ± 5 % humidity, and 12-h light/day cycle (lights on from 7:00 to 19:00) or purchased from National Laboratory Animal Center (Taipei, Taiwan). Mice were housed in four to five per cage. Food and water were available *ad libitum*. Animal handling and drug treatments were performed in strict accordance with the NIH Guide for the Care and Use of Laboratory Animals and approved by the IACUC at Taipei Medical University (LAC-20220033) and Chang Gung University CGU 109–132.

### 2.2 High-fat high-sucrose diet (HFSD)-induced obesity

*Npffr2* Tg mice and WT mice were fed with a rodent diet containing 58 kcal % fat and sucrose (Research Diets, New Brunswick, NJ, USA) or a control chow diet (CD). The body weights of the mice were recorded weekly to monitor their growth curve.

### 2.3 Blood glucose record

Blood glucose levels were recorded every two weeks. Mice were fasted for 6 h with unlimited water access before measurements were taken. Blood glucose levels were measured using a blood glucose meter (Contour Plus, Bayer, Leverkusen, Germany) with blood samples obtained from the end of the tail. Mice were restrained in a restrainer during the measurement.

### 2.4 Food intake monitoring

For the food consumption test, mice were single-housed and fasted for one day before the food intake tests to reduce stress interference. Food intake was measured by weighing the food pellets at indicated time points within 3 h. For daily food and water consumption, the intakes were determined by weighing the food pellets or water containers.

### 2.5 Glucose tolerance test (GTT) and insulin tolerance test (ITT)

The mice were single-housed, fasted with unlimited water access, and left undisturbed for 5–6 h before the test. After measuring basal blood glucose levels, the mice were intraperitoneally injected (IP) with either glucose (2 g/kg) or insulin (0.75 IU/kg; Humulin R, Eli Lilly, Indianapolis, IN, USA), and the level of blood glucose was measured at indicated time points.

### 2.6 RNA extraction and real-time PCR

The RNA extraction and real-time PCR were conducted following previously established protocols [20]. *Gapdh* was utilized as the housekeeping gene. The relative expression levels were quantified by the 2^−ΔΔ^Ct method, and the PCR primer sequences were used as described in the literature [14].

### 2.7 Blood collection and analysis

Blood samples were collected from the facial veins of mice during inhalation anesthesia with 2–2.5 % isoflurane. The samples were clotted for 30 min at room temperature, followed by 15 min of centrifugation at 13,000 rpm to obtain serum. Levels of insulin, triglycerides, cholesterol and non-esterified fatty acid (NEFA) were measured with the commercial colorimetric or ELISA kits following the manufacturer's procedures (Insulin, Mercodia, Sweden; Triglycerides, cholesterol and NEFA, Randox, County Antrim, UK). The homeostasis model assessment-insulin resistance index (HOMA-IR) was acquired by the following formula: (fasting glucose [mg/dL] × fasting insulin [mU/mL]) ÷ 405.

### 2.8 Tissue triglycerides

Triglyceride quantities in brown adipose tissue (BAT) and epididymal white adipose tissue (eWAT) were assessed using commercial colorimetric kits, following the manufacturer's instructions. Results were reported as mg of triglycerides per mg of tissue (Cayman, Ann Arbor, MI, USA).

### 2.9 Tissue collection

Mice were sacrificed, and tissues collected included the mediobasal hypothalamus (MBH), liver, inguinal white adipose tissue (iWAT), eWAT and BAT. The tissues were weighed before storage.

### 2.10 Immunofluorescence staining and hematoxylin and eosin (H&E) staining

The liver, eWAT, and BAT were fixed in formalin. Subsequently, the tissues were embedded in paraffin and sectioned at a thickness of 4 μm. The immunofluorescence and H&E staining were performed following previous protocols [14]. The antibodies utilized include an anti-tumor necrosis factor-alpha (TNFα) antibody (1:100, Abclonal, Woburn, MA, USA), anti-mitochondrial uncoupling protein 1 (UCP1) antibody (1:100, Abclonal), and Cy3-goat-anti-rabbit antibody (Jackson ImmunoResearch, Woburn, MA, USA). Signals were detected using microscopy (Olympus, Tokyo, Japan) and analyzed with ImageJ software (NIH, Bethesda, MD, USA). Liver steatosis was assessed by Dixon's scoring system [1].

### 2.11 Presentation of data and statistics

Data are presented as the mean ± the standard error of the mean (SEM) and were analyzed by two-way AVOVA followed by Bonferroni's multiple comparison tests, three-way ANOVA followed by Tukey's multiple comparison tests, or unpaired Student's t-test. The significance level was set at *p* ≤ 0.05. All analyses were conducted using GraphPad Prism 9 software (GraphPad, Boston, MA, USA).

## 3 Results

### 3.1 Tissue-specific profiles of the NPFF system in Npffr2 overexpression transgenic mice

**Fig. 1:**
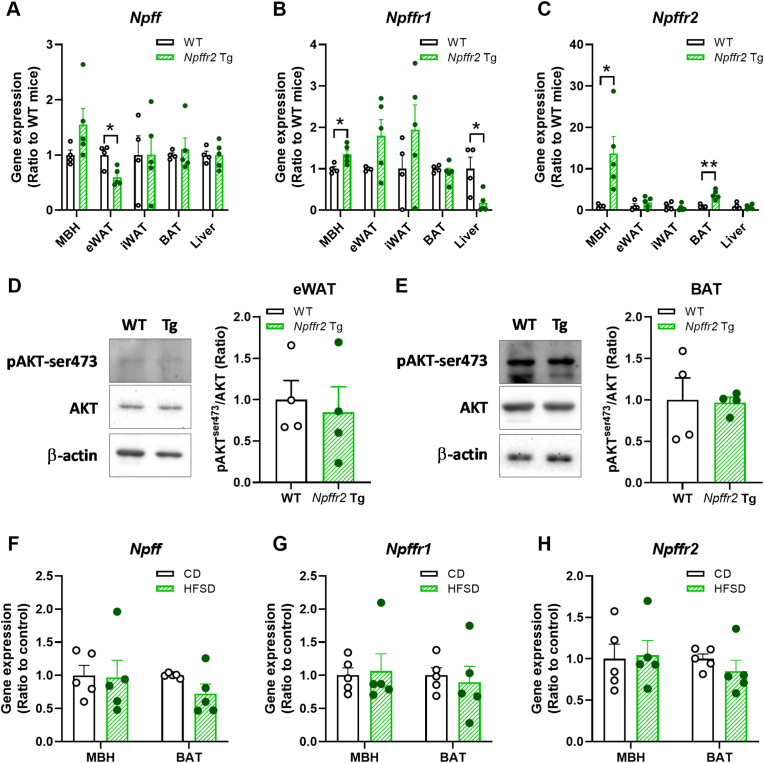
Tissue-specific expression of the NPFF system and AKT signaling in *Npffr2* overexpression transgenic (Tg) mice. Wild-type (WT) and *Npffr2* Tg mice were used to assess tissue-specific expression of NPFF system genes and insulin-induced AKT signaling. (A–C) Gene expression levels of *Npff*, *Npffr1*, and *Npffr2* in the mediobasal hypothalamus (MBH), epididymal white adipose tissue (eWAT), inguinal white adipose tissue (iWAT), brown adipose tissue (BAT), and liver. (D–E) AKT-ser473 phosphorylation in eWAT and BAT of WT and *Npffr2* Tg mice. (F–H) Gene expression levels of *Npff*, *Npffr1*, and *Npffr2* in the MBH and BAT of WT mice fed a chow diet (CD) or high-fat high-sucrose diet (HFSD). Data are presented as mean ± SEM and were analyzed using unpaired Student's t-test. ∗*p* < 0.05, ∗∗*p* < 0.01, compared between indicated groups. N = 4 for WT group and N = 5 for Tg group, except for panels D and E, where sample loss occurred during tissue preparation due to technical issues.

To clarify the tissue-specific impacts of NPFF-NPFFR2 signaling on obesity-related metabolic abnormalities, we first examined the expression of *Npff*, *Npffr1*, and *Npffr2* in the MBH, adipose tissues, and liver of *Npffr2* Tg mice [Fig. 1A-C]. *Npffr2* overexpression in mice significantly upregulated *Npffr2* level in the MBH, while *Npffr1* expression showed a slight increase (less than 50 %), and *Npff* expression remained unchanged. In the WAT and liver of WT mice, *Npffr2* expression was minimal and did not change following overexpression. However, *Npff* expression was reduced in eWAT, and *Npffr1* expression was decreased in the liver of *Npffr2* Tg mice. In contrast, *Npffr2* expression was notably upregulated in BAT of Tg mice. As previously reported, basal AKT-ser473 phosphorylation in the MBH remained unchanged in Tg mice [14]. To determine whether *Npffr2* overexpression alters insulin signaling in peripheral tissues, phosphorylated AKT levels were also examined in WAT and BAT. In the present study, no significant differences were detected between Npffr2 Tg and WT mice [Fig. 1D and E].

Based on previous studies reporting that *Npffr2* is predominantly expressed in the MBH, with lower or minimal expression in BAT, WAT, and liver [9,16], the role of *Npffr2* in diet-induced obesity was further explored using a HFSD feeding model for 15 weeks. However, neither *Npffr2* nor *Npff* and *Npffr1* transcriptional levels were altered following HFSD exposure [Fig. 1F-H].

### 3.2 Npffr2 overexpression promotes greater weight gain in HFSD-induced obesity

**Fig. 2:**
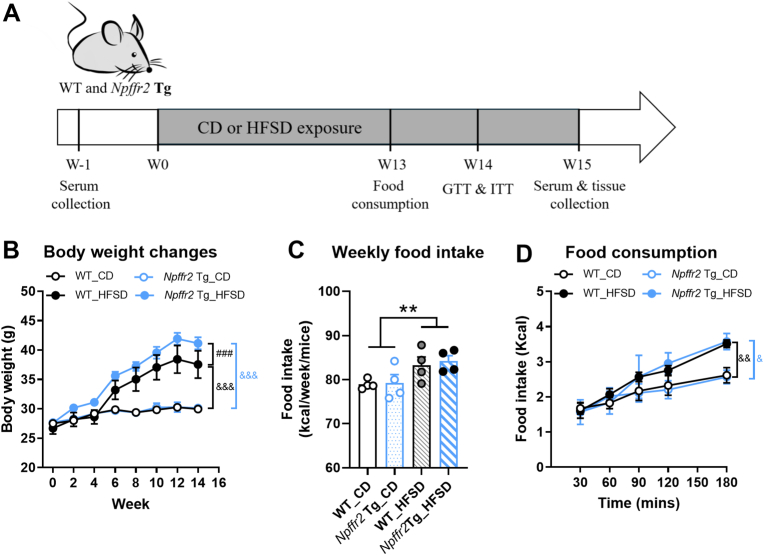
**Diet-induced obesity in *Npffr2* overexpression Tg mice.***Npffr2* Tg mice were fed with HFSD to induce obesity. A series of metabolic parameters were analyzed prior to HFSD feeding (week 0, W0) and before euthanasia (week 15, W15). (A) Flow chart of experimental procedures. (B) The body weight changes. (C) Weekly food intake. (D) Fasting-induced food consumption at week 13. Data are presented as mean ± SEM and were analyzed with three-way ANOVA followed by Tukey's multiple comparison tests (in figure B and D) or two-way ANOVA (in figure C). ^###^*p* < 0.001, compared between WT and *Npffr2* Tg mouse groups within the same diet. ^&^*p* < 0.05, ^&&^*p* < 0.01, ^&&&^*p* < 0.001, compared between CD and HFSD groups within the same genotype. ∗∗*p* < 0.05, compared between CD and HFSD. N = 4 for the WT_CD group and N = 5 for all other groups. Weekly food intake was recorded per cage and averaged per mouse; cage numbers were N = 3 for WT_CD and N = 4 for all other groups.

To investigate whether *Npffr2* overexpression influences susceptibility to diet-induced obesity, WT and *Npffr2* Tg mice were fed with HFSD to induce obesity [Fig. 2A]. No differences in body weight changes were observed between *Npffr2* Tg mice and WT mice in the CD groups. However, *Npffr2* Tg mice exhibited a significantly accelerated body weight gain after exposure to HFSD compared to WT mice [Fig. 2B], despite no differences in food intake within the same dietary groups [Fig. 2C and D].

### 3.3 Npffr2 overexpression exacerbates obesity-induced glucose dysregulation

**Fig. 3:**
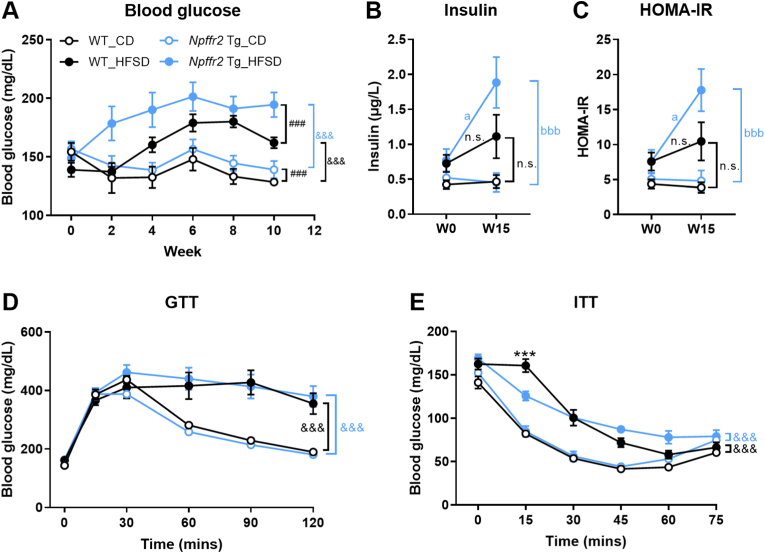
Obesity-altered glucose metabolism in *Npffr2* Tg mice. Parameters related to glucose metabolism were analyzed in *Npffr2* overexpression Tg mic after exposure to HFSD. (A) The blood glucose levels. (B) Serum insulin levels. (C) The Homeostatic Model Assessment for Insulin Resistance (HOMA-IR). (D) The glucose tolerance test (GTT). (E) The insulin tolerance test (ITT). ^###^*p* < 0.001, compared between WT and *Npffr2* Tg mouse groups within the same diet. ^&&&^*p* < 0.001, compared between CD and HFSD groups within the same genotype. ^a^*p* < 0.05, compared between W0 and W15 of WT or *Npffr2* Tg mice within the same diet. ^bbb^*p* < 0.001, compared between CD and HFSD at W15 within the same mouse groups. ∗∗∗*p* < 0.001, compared between WT and *Npffr2* Tg mice at indicated time points of HFSD group. N = 4 for the WT_CD group and N = 5 for all other groups. n.s. = not significant.

Blood glucose levels were elevated after HFSD exposure in both mouse genotypes. *Npffr2* Tg mice exhibited higher blood glucose levels than WT mice in both CD and HFSD groups [Fig. 3A]. Serum insulin levels and HOMA-IR did not significantly increase in WT mice but were significantly elevated in *Npffr2* Tg mice after HFSD feeding [Fig. 3B and C]. No differences in obesity-triggered glucose and insulin intolerance were observed between the two genotypes, as evaluated by GTT and ITT [Fig. 3D and E]. However, 15 min after insulin injection during the ITT, WT mice exhibited slightly higher blood glucose levels compared to *Npffr2* Tg mice [Fig. 3E].

### 3.4 Npffr2 overexpression affects lipid profiles following obesity induction

**Fig. 4:**
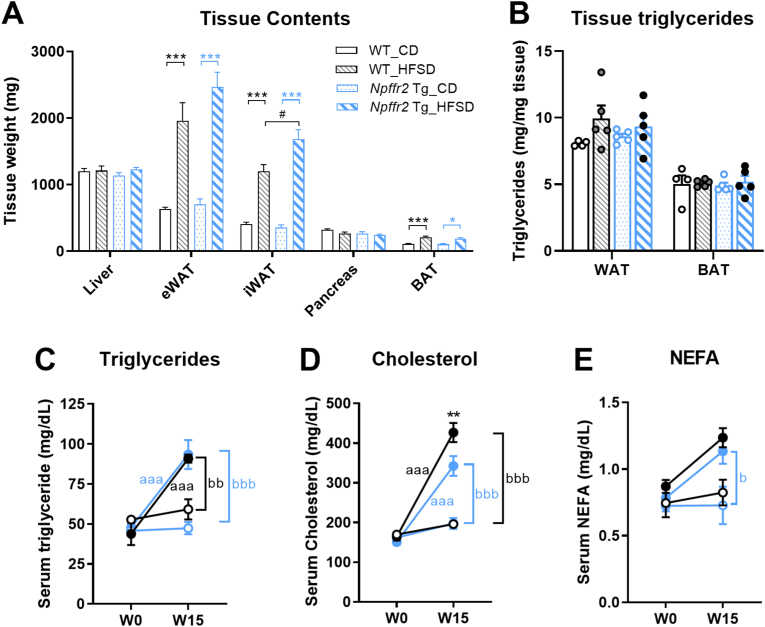
The lipid profiles in WT and *Npffr2* Tg mice after obesity induction. (A) Weights of tissues were measured after mice were euthanized. (B) The levels of triglycerides in WAT and BAT. (C) Serum levels of triglycerides, (D) cholesterol, and (E) non-esterified fatty acid (NEFA) were analyzed at W0 and W15. Data are presented as mean ± SEM. In A and B, data were analyzed with two-way ANOVA followed by Bonferroni's multiple comparison tests. ∗*p* < 0.05, ∗∗∗*p* < 0.001, compared between CD and HFSD groups within the same genotype. ^#^*p* < 0.05, compared between WT and *Npffr2* Tg mouse groups within the same diet. In C-E, data were analyzed with three-way ANOVA followed by Tukey's multiple comparison tests. ^aaa^*p* < 0.001, compared between W0 and W15 of WT or *Npffr2* Tg mice within the same diet. ^b^*p* < 0.05, ^bb^*p* < 0.01, ^bbb^*p* < 0.001, compared between CD and HFSD at W15 within the same mouse groups. ∗∗*p* < 0.01, compared between WT and *Npffr2* Tg mice at W15 of HFSD group. N = 4 for the WT_CD group and N = 5 for all other groups.

Upon tissue weight measurement, both groups of mice showed increased adipose tissue mass following HFSD feeding [Fig. 4A]. Notably, *Npffr2* Tg mice exhibited a significantly greater increase in iWAT weight compared to WT mice after HFSD exposure. However, triglyceride levels in both WAT and BAT did not differ between WT and *Npffr2* Tg mice [Fig. 4B]. Circulating triglycerides and cholesterol levels increased in both WT and *Npffr2* Tg mice after 15 weeks of HFSD feeding, with *Npffr2* Tg mice exhibiting a slightly lower increase in serum cholesterol levels compared to WT mice [Fig. 4C and D]. On the other hand, circulating NEFA levels in WT mice showed no statistical differences between the two diets, whereas in *Npffr2* Tg mice, the HFSD group exhibited a significant increase in NEFA levels compared to the CD control [Fig. 4E].

**Fig. 5:**
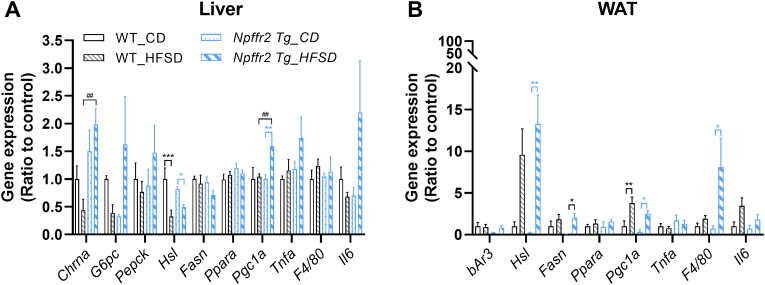
The gene changes in the liver and WAT of WT and *Npffr2* Tg mice after obesity induction. The transcriptional levels of the indicated genes in the (A) liver and (B) WAT after mice obesity induction in mice. Data are presented as mean ± SEM and were analyzed with two-way ANOVA followed by Bonferroni's multiple comparison tests. ∗*p* < 0.05, ∗∗*p* < 0.01, ∗∗∗*p* < 0.001, compared between CD and HFSD groups within the same genotype. ^##^*p* < 0.01, compared between WT and *Npffr2* Tg mouse groups within the same diet. N = 4 for the WT_CD group and N = 5 for all other groups.

Transcriptomes related to gluconeogenesis, lipid metabolism, and inflammation were analyzed in the liver and WAT. In the liver, the mRNA level of the cholinergic receptor nicotinic alpha (*Chrnα*, which receives acetylcholine from the vagus nerve and participates in energy metabolism, such as hepatic glucose production [21]) did not differ after obesity induction in WT mice. In contrast, *Npffr2* Tg mice fed with HFSD showed elevated *Chrnα* expression compared to the CD control [Fig. 5A]. The gene levels of rate-limiting enzymes in hepatic gluconeogenesis, glucose-6-phosphatase (*G6pc*) and phosphoenolpyruvate carboxykinase (*Pepck*), did not change within the groups [Fig. 5A]. Both WT and *Npffr2* Tg mice exhibited downregulated hepatic hormone-sensitive lipase (*Hsl*, key enzyme involved in lipolysis) levels in the HFSD groups compared to the CD control, while no differences were observed in fatty acid synthase (*Fasn*, key enzyme in lipogenesis) levels [Fig. 5A]. Among the fatty acid oxidation-related genes, peroxisome proliferator-activated receptor α (*Pparα*, transcriptional regulator of fatty acid β-oxidation) showed no difference within the groups, whereas peroxisome proliferator-activated receptor gamma coactivator-1 α (*Pgc1a*, promotes β-oxidation and mitochondrial biogenesis) was upregulated in *Npffr2* Tg mice after HFSD exposure compared to both CD-fed *Npffr2* Tg mice and HFSD-fed WT mice [Fig. 5A]. Among the genes of pro-inflammatory cytokines (*Tnfa*, *Il6*) and inflammation-related genes *(F4/80*, macrophage marker), no alterations were observed between the groups [Fig. 5A]. In WAT, mRNA level of β3 adrenergic receptor (*bAr3,* which regulate lipid metabolism in response to central sympathetic stimulation) did not differ between WT and *Npffr2* Tg mice. On the other hand, *Npffr2* Tg exhibited upregulated *Hsl* and *Fasn* levels after HFSD feeding compared to the CD control, while no changes were observed in WT groups [Fig. 5B]. The *Ppara* gene level did not differ between WT and *Npffr2* Tg mice, while *Pgc1a* was significantly upregulated in the HFSD groups of both WT and *Npffr2* Tg mice [Fig. 5B]. Regarding pro-inflammatory cytokines, the transcriptional levels of *Tnfa* and *Il6* did not differ between WT and *Npffr2* Tg mice. However, while *F4/80* levels remained unchanged between CD-fed and HFSD-fed WT mice, HFSD-fed *Npffr2* Tg mice exhibited significantly upregulated *F4/80* level compared to CD-fed *Npffr2* Tg mice [Fig. 5B].

### 3.5 Npffr2 overexpression affects metabolic dysregulation in peripheral tissues following the induction of obesity

**Fig. 6:**
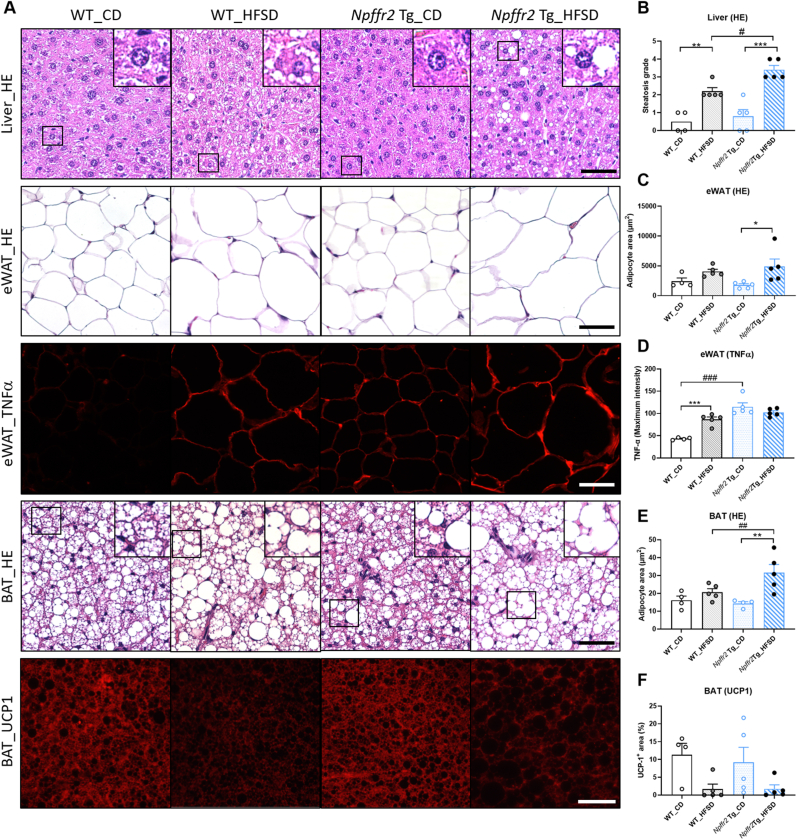
Lipid accommodations and protein expressions after HFSD-induced obesity in WT and *Npffr2* Tg mice. (A) The represented images of H&E staining and immunofluorescence staining of indicated tissues. (B) The steatosis grade of liver sections. (C) The sizes of adipocytes in eWAT. (D) The protein levels of TNFα in eWAT (presented in signal intensity). (E) The sizes of adipocytes in BAT. (F) The protein levels of UCP1 in BAT (presented as UCP1-positive area in %). Data are presented as mean ± SEM and were analyzed with two-way ANOVA followed by Bonferroni's multiple comparison tests. ∗*p* < 0.05, ∗∗*p* < 0.01, ∗∗∗*p* < 0.001, compared between CD group and HFSD group within the same genotype. ^#^*p* < 0.05, ^##^*p* < 0.01, ^###^*p* < 0.001, compared between WT and *Npffr2* Tg mouse groups within the same diet. N = 4 for the WT_CD group and N = 5 for all other groups. Scale bar = 20 μm. Image quantification was based on 5 individual fields per mouse.

Morphological changes in peripheral tissues were assessed using H&E staining and immunohistochemistry staining [Fig. 6A]. Hepatic steatosis remarkably increased following obesity induction in both WT and *Npffr2* Tg mice, with a more pronounced increase in *Npffr2* Tg mice after HFSD feeding compared to WT mice [Fig. 6B]. Moreover, the size of adipocytes in eWAT remained unchanged in WT mice after HFSD exposure, but was significantly enlarged in HFSD-fed *Npffr2* Tg mice compared to the CD control [Fig. 6C]. Interestingly, the pro-inflammatory cytokine TNFα increased in eWAT of WT mice after HFSD feeding, and was also elevated in CD-fed *Npffr2* Tg mice compared to CD-fed WT mice. However, the increase in *Npffr2* Tg mice was not further amplified after HFSD feeding [Fig. 6D]. Similar to eWAT, the size of adipocytes in BAT remained unchanged in WT groups but was enlarged in *Npffr2* Tg mice after HFSD feeding. The size of BAT adipocytes was significantly larger in HFSD-fed *Npffr2* Tg mice compared to HFSD-fed WT mice [Fig. 6E]. The expression of the thermogenesis protein UCP1 in BAT showed no differences between the genotypes [Fig. 6F].

## 4 Discussion

In this study, *Npffr2* overexpression aggravated obesity-associated metabolic abnormalities, particularly in glucose regulation and lipid handling across liver and adipose tissues. The results underscore the adverse impact of *Npffr2* overexpression on metabolic health.

The functional impact of *Npffr2* overexpression depends on the availability of endogenous ligands. Although no significant change in *Npff* expression was observed in the MBH in this study, previous reports have shown increased hypothalamic NPFF levels in Tg mice [19]. This discrepancy may reflect differences in tissue sampling or individual variability. Notably, NPFFR2 also exhibits binding affinity for other RF-amide peptides, such as NPAF, prolactin-releasing peptide (PrRP), RFamide-related peptide (RFRP-3, also known as neuropeptide VF), kisspeptin, and 26RFa. Therefore, the influence of alternative presynaptic ligands cannot be excluded [[22], [23], [24]]. Moreover, it is plausible that basal levels of NPFF were sufficient to activate the upregulated receptors under HFSD-induced metabolic stress.

The metabolic outcomes of NPFFR2-deficient mice have been reported under both CD and HFD feeding conditions [15]. The study demonstrated reduced body weight gain, increased energy intake, improved plasma lipid profiles, as well as the development of glucose intolerance and insulin resistance. NPFFR2 deficiency was also associated with either improvement or worsening of hepatic steatosis and metabolic parameters, depending on nutritional status and sex. Although the findings are not entirely consistent within or across studies, including our own, these observations highlight the important role of NPFFR2 in regulating energy metabolism under different nutritional conditions.

One important question is how NPFFR2 exerts its influence across different organs or tissues implicated in obesity-associated metabolic disturbances. *Npffr2* is known to be predominantly expressed in the hypothalamus, a key region involved in the regulation of systemic metabolism [8,12,17,25]. In contrast, *Npffr2* expression was found to be extremely low in adipose tissues, liver, and skeletal muscle, consistent with previous reports [9,16]. The minimal expression of *Npffr2* in these peripheral tissues of WT mice suggests that its role in these regions may be limited or secondary compared to its central actions. In our model, *Npffr2* overexpression was driven by the neuron-specific enolase (NSE) promoter [19]. As expected, *Npffr2* expression was markedly elevated in the MBH of *Npffr2* Tg mice, while no significant changes were detected in WAT and liver compared to WT controls. However, an unexpected peripheral leakage was observed in BAT. This ectopic expression may be attributable to the influence of the H19 insulator element included in the transgene construct, as discussed in our previous report [19]. Notably, the increase in BAT was relatively modest compared to the MBH. Furthermore, since effective NPFFR2 signaling depends on presynaptic ligand release, the nonspecific overexpression in BAT is likely to have minimal functional impact in the current study. Our previous findings showed that NPFF-NPFFR2 signaling negatively regulates central insulin sensitivity. Specifically, overexpression of *Npffr2* in the ARC exacerbated metabolic disturbances under T2DM conditions, while deletion of *Npffr2* attenuated T2DM-induced metabolic abnormalities [14]. In the current study, insulin signaling, as assessed by AKT phosphorylation, remained unchanged in WAT and BAT of *Npffr2* Tg mice compared to WT mice. These findings suggest that NPFFR2 regulates metabolic function primarily through central mechanisms, with limited contribution from peripheral tissues. This is further supported by previous studies indicating that NPFF and its receptors act centrally to influence energy homeostasis [13,16,17,26]. Based on these findings, we hypothesize that the metabolic effects of NPFFR2 observed in the current study are mediated through the impairment of central insulin signaling.

NPFF has been reported to regulate food intake, potentially through central mechanisms [26]. NPFF was also reported to suppress orexigenic NPY neuron activity in the human ARC, emphasizing the involvement of NPFF in the regulation of feeding behaviors [17]. The high colocalization of NPFFR2 with NPY- and AGRP-expressing neurons in the human brain further highlights the role of NPFFR2 in regulating feeding behaviors [17]. In our previous study, *Npffr2* Tg mice exhibited diminished insulin-induced suppression of food intake [14], suggesting a potential role in appetite regulation. However, no significant differences in food intake were observed between WT and *Npffr2* Tg mice following HFSD feeding. These findings imply that the effect of NPFFR2 on feeding behavior may depend on metabolic state, and that peripheral signals (e.g., from adipose tissue, liver, and gut) may counterbalance central regulation [27].

Despite unchanged food intake and comparable physical activity [24], *Npffr2* overexpression mice showed marked weight gain and adipose expansion, consistent with previous reports showing reduced weight gain in *Npffr2* knockout mice under HFD [15]. This dissociation between food intake and weight gain is consistent with previous findings [16] and raises the possibility that NPFFR2 may influence energy balance through mechanisms other than feeding behavior, such as altered metabolic efficiency or energy expenditure. However, further functional studies are needed to substantiate this hypothesis.

At the tissue and molecular levels, gene expression analysis indicated no significant changes in lipogenesis- and lipolysis-related genes in WAT between genotypes. However, an increase in F4/80 expression (which reflects the extent of macrophage infiltration) and elevated TNF-α protein levels in WAT imply an enhanced inflammatory state, which may promote abnormal lipid accumulation and adipocyte hypertrophy. In addition, whitening of BAT was observed in *Npffr2* Tg mice, potentially reflecting altered thermogenic function, although UCP1 expression remained unchanged and no direct measurements of thermogenesis were performed. In the liver, lipolytic gene (*Hsl*) and lipogenesis gene (*Fans*) expression remained unchanged. However, more pronounced hepatic steatosis was observed, which may reflect impaired lipid clearance or utilization. Notably, the hepatic expression of *Chrna* was increased, indicating enhanced signaling from the central nervous system in *Npffr2* Tg mice. The upregulation of *Pgc1a* in the liver may reflect a compensatory response. However, these changes appeared insufficient to prevent excessive lipid accumulation. These findings may contribute to the increased adipose tissue inflammation, which may disrupt metabolic homeostasis, driving energy storage toward fat deposition rather than energy expenditure.

To better characterize the inflammatory phenotype, we acknowledge that while the elevated TNF-α and F4/80 levels imply increased adipose tissue inflammation, these markers alone are insufficient to fully define immune polarization. This limitation may partly explain the apparent contrast between our findings and previous reports showing that NPFFR2 activation in ATMs promotes anti-inflammatory polarization [18]. Additionally, previous studies have also noted that NPFFR2 expression in ATMs and circulating NPFF levels are reduced under metabolic stress conditions [18]. This inconsistency may reflect differences in the location and context of NPFFR2 signaling. One important distinction lies in where and how NPFFR2 is activated. While earlier studies focused on cell-specific regulation within ATMs, our model involves *Npffr2* overexpression in the hypothalamus, which may indirectly influence immune balance in adipose tissue by altering central metabolic signals, sympathetic activity, or neuroendocrine outputs. Thus, although NPFFR2 signaling in local macrophages may have anti-inflammatory effects, prolonged central NPFFR2 activation could override this benefit and promote peripheral inflammation through neuroendocrine or autonomic pathways. Moreover, the effects of NPFF signaling may vary by tissue compartment. The reduced NPFFR2 expression in ATMs after prolonged HFD exposure could reflect a compensatory downregulation under chronic metabolic stress [18]. Of note, our data also show elevated *Npffr2* expression in BAT of *Npffr2* Tg mice. Given that previous studies have demonstrated that NPFF promotes mitophagy, thereby facilitating the transition of beige adipose tissue into WAT [28], we cannot rule out the possibility that NPFFR2 may exert a direct effect in BAT that contributes to tissue whitening. Together, these findings highlight the complex and context-dependent role of NPFFR2 in regulating both metabolic and immune responses, with contributions from both central and peripheral mechanisms.

In our study, *Npffr2* overexpression in mice fed a HFSD led to the development of insulin resistance, as indicated by elevated fasting insulin levels and an increased HOMA-IR index, despite no significant changes in glucose or insulin tolerance tests. The lack of significant differences in GTT and ITT results may reflect early or compensatory stages of insulin resistance. During the initial phase, pancreatic β-cells often increase insulin secretion to maintain normal glucose tolerance. The observed elevations in fasting insulin and HOMA-IR values support this compensatory mechanism. These findings suggest that *Npffr2* overexpression may contribute to an early insulin-resistant state, potentially progressing to overt metabolic dysfunction with prolonged dietary stress. While group sizes were limited due to constraints in transgenic mouse breeding, the consistent elevations in fasting insulin and HOMA-IR suggest sufficient to detect early metabolic changes, likely without masking differences in GTT or ITT.

This insulin resistance observed in the study contrasts with a recent report, which showed that *Npffr2* deficiency worsens glucose intolerance in HFD-fed mice, suggesting a protective role for NPFFR2 in glucose handling [15]. However, another study demonstrated that *Npff* deficiency improved glucose tolerance without affecting insulin excursions, which aligns more closely with our findings and supports a potentially adverse role for NPFF signaling in glucose regulation [13]. Although glucose tolerance appeared unaffected in our model, the observed hyperinsulinemia implies a state of compensatory insulin resistance. This is consistent with emerging evidence that NPFF interferes with insulin-related pathways. For instance, NPFF is degraded by insulin-degrading enzyme, and this process is suppressed in the presence of insulin [29]. Additionally, NPFF has been shown to inhibit glucose- and arginine-stimulated insulin secretion [30]. Together, these findings suggest that NPFFR2 signaling may influence insulin regulation differently depending on metabolic conditions, potentially contributing to insulin resistance.

This study is the first to utilize an *Npffr2* gain-of-function mouse model to investigate the metabolic impact of chronic NPFFR2 activation, offering a novel perspective that complements previous loss-of-function or region-specific studies. By enabling the assessment of systemic consequences driven by sustained NPFFR2 signaling, this model better mimics potential pharmacological compounds and provides valuable insights into metabolic dysregulation relevant to future NPFFR2-targeted drug development.

## 5 Conclusions

In conclusion, this study provides descriptive evidence that NPFFR2 overexpression, particularly within the hypothalamus, may contribute to obesity-associated alterations in body composition, glucose and lipid metabolism, and inflammatory responses. Although previous studies have implicated NPFFR2 in central insulin regulation, the present work extends these findings by characterizing its broader metabolic effects under chronic dietary stress. Given the absence of region-specific functional manipulations in this model, further mechanistic studies are needed to clarify the specific pathways and tissue compartments through which NPFFR2 modulates metabolic homeostasis. These insights may help evaluate the potential of NPFFR2 as a therapeutic target in metabolic disorders.

## Declaration of competing interest

The authors declare that they have no competing interests.

## Data availability statement

The datasets generated or analyzed during the current study are available from the corresponding author on reasonable request.

## Funding

This work was supported by the 10.13039/100020595National Science and Technology Council, Taiwan [NSTC 110-2326-B-038-002-MY3 and NSTC 113-2320-B-038-017]. The funding sources had no involvement in the research conduct.
